# Colorectal cancer risk variants at 8q23.3 and 11q23.1 are associated with disease phenotype in *APC* mutation carriers

**DOI:** 10.1007/s10689-016-9877-5

**Published:** 2016-02-15

**Authors:** Z. Ghorbanoghli, M. H. Nieuwenhuis, J. J. Houwing-Duistermaat, S. Jagmohan-Changur, F. J. Hes, C. M. Tops, A. Wagner, C. M. Aalfs, S. Verhoef, E. B. Gómez García, R. H. Sijmons, F. H. Menko, T. G. Letteboer, N. Hoogerbrugge, T. van Wezel, H. F. A. Vasen, J. T. Wijnen

**Affiliations:** 1 Netherlands Foundation for the Detetion of Hereditary Tumors, Leiden, The Netherlands; 2 Department of Gastroenterology and Hepatology, Leiden University Medical Centre, Rijnsburgerweg 10, 2333 AA Leiden, The Netherlands; 3 Department of Medical Statistics and Bioinformatics, Leiden University Medical Center, Leiden, The Netherlands; 4 Department of Human Genetics, Leiden University Medical Center, Leiden, The Netherlands; 5 Department of Clinical Genetics, Leiden University Medical Center, Leiden, The Netherlands; 6 Department of Clinical Genetics, Erasmus Medical Center, Rotterdam, The Netherlands; 7 Department of Clinical Genetics, Amsterdam Medical Centre, Amsterdam, The Netherlands; 8 Family Cancer Clinic, the Netherlands Cancer Institute, Amsterdam, The Netherlands; 9 Department of Clinical Genetics, University of Maastricht, Maastricht, The Netherlands; 10 Department of Genetics, University Medical Centre Groningen, University of Groningen, Groningen, The Netherlands; 11 Department of Clinical Genetics, VU University Medical Center, Amsterdam, The Netherlands; 12 Department of Medical Genetics, University Medical Centre Utrecht, Utrecht, The Netherlands; 13 Department of Human Genetics, Radboud University Nijmegen Medical Center, Nijmegen, The Netherlands; 14 Departments of Pathology, Leiden University Medical Center, Leiden, The Netherlands

**Keywords:** Familial adenomatous polyposis, Cancer genetics, Colonic adenomas, Genetic polymorphisms

## Abstract

Familial adenomatous polyposis (FAP) is a dominantly inherited syndrome caused by germline mutations in the *APC* gene and characterized by the development of multiple colorectal adenomas and a high risk of developing colorectal cancer (CRC). The severity of polyposis is correlated with the site of the *APC* mutation. However, there is also phenotypic variability within families with the same underlying *APC* mutation, suggesting that additional factors influence the severity of polyposis. Genome-wide association studies identified several single nucleotide polymorphisms (SNPs) that are associated with CRC. We assessed whether these SNPs are associated with polyp multiplicity in proven *APC* mutation carriers. Sixteen CRC-associated SNPs were analysed in a cohort of 419 *APC* germline mutation carriers from 182 families. Clinical data were retrieved from the Dutch Polyposis Registry. Allele frequencies of the SNPs were compared for patients with <100 colorectal adenomas versus patients with ≥100 adenomas, using generalized estimating equations with the *APC* genotype as a covariate. We found a trend of association of two of the tested SNPs with the ≥100 adenoma phenotype: the C alleles of rs16892766 at 8q23.3 (OR 1.71, 95 % CI 1.05–2.76, *p* = 0.03, dominant model) and rs3802842 at 11q23.1 (OR 1.51, 95 % CI 1.03–2.22, *p* = 0.04, dominant model). We identified two risk variants that are associated with a more severe phenotype in *APC* mutation carriers. These risk variants may partly explain the phenotypic variability in families with the same *APC* gene defect. Further studies with a larger sample size are recommended to evaluate and confirm the phenotypic effect of these SNPs in FAP.

## Introduction

Familial adenomatous polyposis (FAP) is a hereditary colorectal cancer (CRC) susceptibility syndrome, caused by germline mutations in the adenomatous polyposis coli (*APC*) gene, which is located on chromosome 5. Carriers of mutations in the *APC* gene develop multiple colorectal adenomas and consequently have a high risk of developing CRC. The risk of CRC in these individuals is related to the number of colorectal adenomas [1]. The severity of polyposis, reflected by the number of colorectal adenomas and the age of onset, is correlated with the site of the *APC* mutation [2]. Most patients with mutations in the codon 1250–1464 region develop thousands of colorectal adenomas in the first or second decades of life. Patients with a mutation at either end or in a specific splice site region of the *APC* gene (codons <157, 312–412, >1595) usually have an attenuated polyposis phenotype, with less than a hundred polyps and an age of onset in the third or fourth decades. The majority of FAP patients have mutations in the remainder of the gene and develop hundreds to thousands of polyps from the second decade of life onwards. However, there is also phenotypic variability within FAP families with the same underlying gene defect, suggesting that beside the *APC* genotype, other factors also play a role in determining the severity of polyposis and the risk of CRC.

Both environmental and genetic factors are known to influence CRC risk [3]. To date, several single nucleotide polymorphisms (SNPs) that show an association with sporadic CRC have been identified by genome-wide association studies (GWAS) [4–10]. Furthermore, gene-environmental interactions may play a role in the effect of SNPs on CRC predisposition [11].

Two of these CRC-associated SNPs (rs16892766 and rs3802842) have been shown to be significantly associated with the risk of CRC and/or age of CRC development in patients with Lynch syndrome [12–14].

We hypothesized that SNPs associated with sporadic CRC may play a role in polyp formation in patients with a germline *APC* mutation. In the present study, we assessed whether known CRC-associated SNPs influence the disease phenotype in patients with a germline *APC* mutation.

## Methods

### Patients

A total of 419 patients from 182 families with a proven germline *APC* mutation were selected from the polyposis database of the Netherlands Foundation for the Detection of Hereditary Tumors. All patients gave informed consent for registration in the database and for use of their medical data for research purposes. All patients had also given written consent for use of their DNA in further institutional ethics-approved research into their condition before the study. The following data were collected: gender, mode of diagnosis (symptomatic or by screening), age at diagnosis of polyposis and CRC, cumulative number of colorectal adenomas, age at colorectal surgery, date and status of last follow-up. Based on the *APC* mutation site, patients were categorized into attenuated, intermediate or severe genotype groups, as described in the introduction [2].

### Genotyping of SNPs

DNA was extracted from peripheral lymphocytes using an automated procedure (Gentra Systems, Minneapolis, USA) and quantified using Picogreen (Invitrogen, California, USA). Genotyping of the SNPs was performed with the KASPar genotyping system, and outsourced to KBioscience (http://www.kbioscience.co.uk).

### Statistical analysis

The Hardy–Weinberg equilibrium of the SNPs was first tested using PLINK, version 1.07 [15]. Further analyses were performed using PASW Statistics 20. The patients were categorized according to the number of colorectal adenomas. We defined two groups: the first group with less than 100 adenomas, and the second group with 100 or more adenomas. The allele frequency of the SNPs was compared between the two groups. To assess association between phenotype and SNP, genotypic odds ratios (OR) and 95 % confidence intervals (CI) were computed using the Generalized Estimating Equation, with exchangeable as working covariance structure for observations within families. A general model for the risk alleles was used for assessing statistical significance, where a dominant model was used in case of rare alleles. As a second step, we also fitted dominant and recessive models to provide further information. For testing, Wald tests were applied. *APC* mutation site, categorized as genotype group, was included in the model as a covariate. For all statistical analysis, a *p* value of <0.05 was considered to show a trend of association. When Bonferroni multiple testing correction was applied for 15 SNPs at thirteen susceptibility loci, *p* < 0.004 should be considered as cut off point for significance.

## Results

A total of 419 *APC* mutation-positive patients were included, of which 188 (44.9 %) had more than 100 colorectal adenomas. The clinical and demographic characteristics of the study subjects are shown in Table 1.

**Table 1:** Clinical and demographic characteristics of 419 *APC* mutation carriers

	<100 adenomas (N = 231)	≥100 adenomas (N = 188)
Gender		
Male (%)	111 (48 %)	99 (53 %)
Polyposis		
Mean age at diagnosis, years	26.5	27.6
Mode of diagnosis		
Symptomatic (%)	34 (15 %)	72 (38 %)
Screening (%)	197 (85 %)	116 (62 %)
CRC (%)	19 (8 %)	30 (16 %)
Mean age at CRC, years (range)	43.4	40.4
Mutation group		
Attenuated (%)	50 (22 %)	20 (11 %)
Intermediate (%)	172 (74 %)	141 (75 %)
Severe (%)	9 (4 %)	27 (14 %)
Last follow-up		
Age, years	34.7	40.4
Status at last follow-up		
Alive (%)	221 (96 %)	165 (88 %)
Dead due to CRC (%)	9 (4 %)	14 (7 %)
Dead due to other cause (%)	1 (0.4 %)	9 (5 %)

Regarding differences between groups, more patients with >100 colorectal adenomas (38 %) were symptomatic on diagnosis compared to the other group (15 %). In addition, the frequency of CRC in the >100 adenoma group was significantly higher than the other group. About 75 % of patients from both phenotype groups had an intermediate phenotype but the proportion of patients with mutations belonging to the attenuated genotype group was twice as high in <100 adenoma as the >100 adenoma group (Table 1).

Of the 16 SNPs tested, fifteen SNPs were in Hardy–Weinberg equilibrium (Table 2). One SNP, rs4939827, showed borderline significant deviance and was excluded from further analyses.

**Table 2:** Test for Hardy–Weinberg equilibrium

SNP	Chromosome region	Alleles major/minor	Risk allele	HWE P value	MAF^a^ (allele)	Gene	Reference
rs6691170	1q41	G/T	T	0.2182	0.321 (T)	*DUSP10*	[9]
rs6687758	1q41	A/G	G	0.1461	0.160 (G)	*DUSP10*	[9]
rs10936599	3q26.2	C/T	C	0.8902	0.229 (T)	*MYNN*	[9]
rs16892766	8q23.3	A/C	C	0.5592	0.091 (C)	*EIF3H*	[4]
rs6983267	8q24.21	G/T	G	0.2798	0.461 (T)	*MYC*	[5]
rs10795668	10p14	G/A	G	0.1723	0.311 (A)	Unknown	
rs3802842	11q23.1	A/C	C	0.6216	0.265 (C)	*POU2AF1*	[6]
rs7136702	12q13.13	C/T	T	0.8298	0.346 (T)	*LARP4*	[9]
rs11169552	12q13.13	C/T	C	0.6966	0.247 (T)	*DIP2B*	[9]
rs4444235	14q22.2	T/C	C	0.2362	0.432 (C)	*BMP4*	[7]
rs4779584	15q13.3	C/T	T	1	0.159 (T)	*GREM1*	[8]
rs9929218	16q22.1	G/A	G	0.4207	0.304 (A)	*CDH1*	[7]
rs4939827	18q21.1	C/T	T	0.04911	0.435 (T)	*SMAD7*	[10]
rs10411210	19q13.11	C/T	C	0.07355	0.127 (T)	*RHPN2*	[7]
rs961253	20p12.3	C/A	A	0.1397	0.311 (A)	*BMP2*	[7]
rs4925386	20q13.33	C/T	C	0.4955	0.311 (T)	*LAMA5*	[9]

^a^Minor allele frequency (MAF) in patients included in this study

**Fig. 1:**
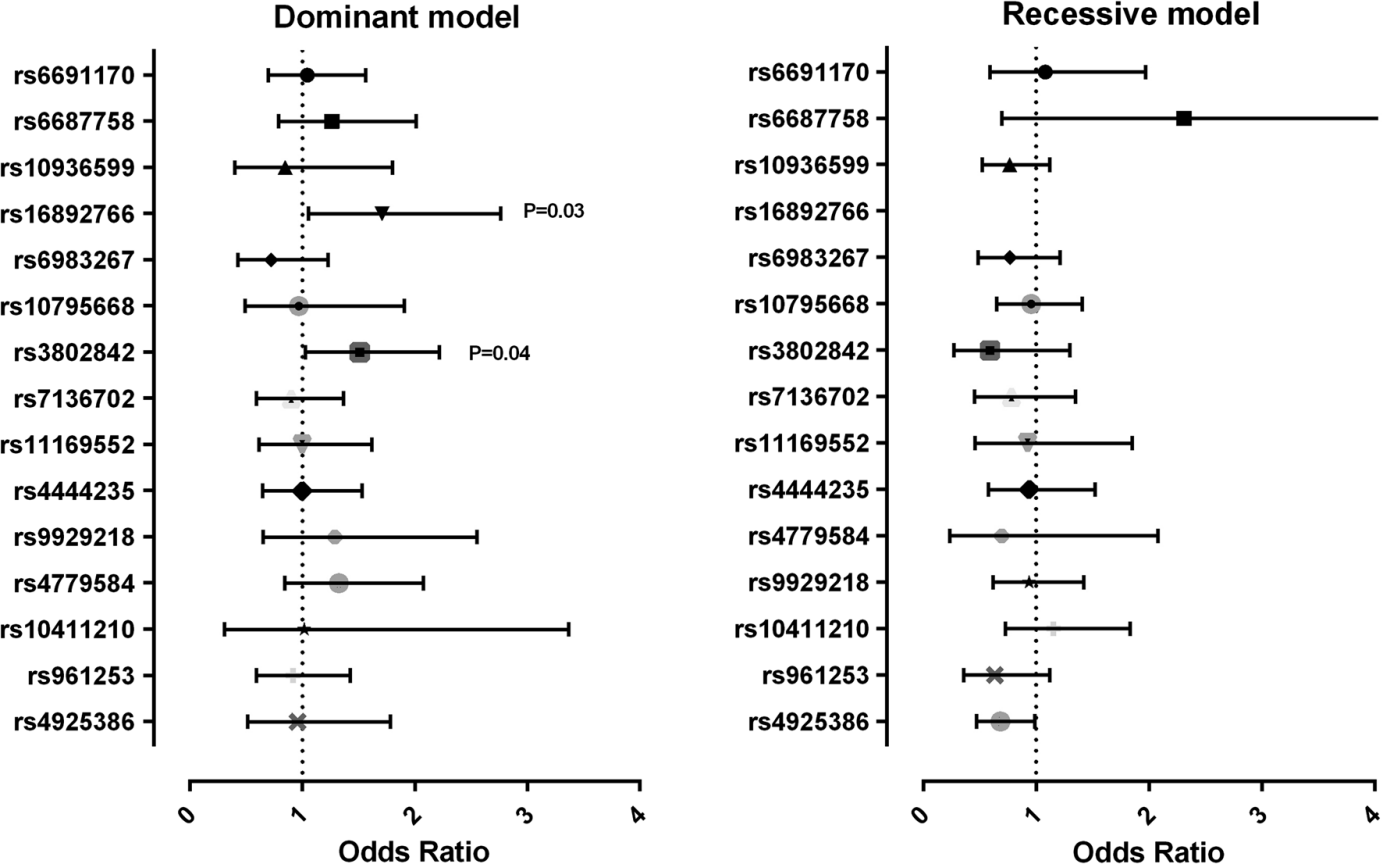
Forest plot: results for 15 susceptibility SNPs in patients with ≥100 polyps and <100 polyps, under recessive and dominant inheritance models

The association of all 15 SNPs with disease phenotype in *APC* mutation carriers was modelled by Generalized Estimating Equilibrium with exchangeable variance structure. Allelic distribution, genotypic ORs and the corresponding 95 % CIs for each SNP are shown in Table 3 (general inheritance model) and Fig. 1 (dominant and recessive inheritance models). Due to the low number of patients with the CC genotype for rs16892766, the genotypic OR for the CC could not be estimated and therefore the dominant model was applied.

**Table 3:** Results for 15 CRC susceptibility SNPs in patients with ≥100 polyps and <100 polyps, under a codominant inheritance model

SNP	Chromosome position	Genotype	Total (%)	≥100 polyps (%)	Odds ratio	95 % CI	*p* value Wald 1 df	*p* value Wald 2 df
rs6691170	1q41		410 (100)	182				0.96
		GG	195 (47.6)	86 (47.3)	1			
		TG	167 (40.7)	74 (40.7)	1.03	0.68–1.56	0.89	
		TT	48 (11.7)	22 (12.0)	1.01	0.57–2.13	0.78	
rs6687758	1q41		419 (100)	188				0.35
		AA	300 (71.6)	133 (70.7)	1			
		GA	104 (24.8)	46 (24.5)	2.42	0.71–1.87	0.56	
		GG	15 (3.6)	9 (4.8)	2.10	0.72–8.17	0.16	
rs10936599	3q26.2		410 (100)	180				0.39
		TT	21 (5.1)	10 (5.5)	1			
		TC	146 (35.6)	68 (37.8)	0.99	0.46–2.13	0.98	
		CC	243 (59.3)	102 (56.7)	0.76	0.35–1.65	0.49	
rs16892766	8q23.3		417 (100)	187				
		AA	343 (82.3)	146 (78.1)	1			
		CA and CC^a^	74 (17.7)	41 (21.9)	1.71	1.05–2.76	**0.03**	
		[CC]	[2]	[2]				
rs6983267	8q24.21		408 (100)	179				0.32
		TT	92 (22.5)	45 (25.1)	1			
		TG	192 (47.1)	84 (46.9)	0.77	0.43–1.40	0.40	
		GG	124 (30.4)	50 (27.9)	0.64	0.36–1.14	0.13	
rs10795668	10p14		417 (100)	187				0.98
		AA	33 (7.9)	14 (7.5)	1			
		GA	193 (46.3)	85 (45.4)	0.99	0.49–1.98	0.97	
		GG	191 (45.8)	88 (47.1)	0.95	0.46–1.94	0.88	
rs3802842	11q23.1		415 (100)	185				**0.02**
		AA	226 (54.5)	91 (49.2)	1			
		CA	158 (38.1)	84 (45.4)	1.70	1.13–2.55	**0.01**	
		CC	31 (7.5)	10 (5.4)	0.76	0.34–1.68	0.49	
rs7136702	12q13.13		413 (100)	185				0.65
		CC	175 (42.4)	82 (44.3)	1			
		TC	190 (46.0)	84 (45.4)	0.94	0.60–1.46	0.78	
		TT	48 (11.6)	19 (10.3)	0.75	0.42–1.37	0.35	
rs11169552	12q13.13		415 (100)	185				0.97
		CC	237 (57.1)	106 (57.3)	1			
		TC	151 (36.4)	68 (36.8)	1.01	0.61–1.68	0.97	
		TT	27 (6.5)	11 (5.9)	0.93	0.45–1.93	0.84	
rs4444235	14q22.2		415 (100)	184				0.97
		TT	128 (30.8)	57 (31.0)	1			
		CT	215 (51.8)	94 (51.1)	1.01	0.65–1.58	0.96	
		CC	72 (17.3)	33 (17.9)	0.95	0.53–1.69	0.85	
rs4779584	15q13.3		411 (100)	183				0.27
		CC	290 (70.6)	123 (67.2)	1			
		CT	111 (27.0)	57 (31.1)	1.77	0.60–5.22	0.30	
		TT	10 (2.4)	3 (1.6)	1.28	0.42–3.86	0.67	
rs9929218	16q22.1		415 (100)	186				0.66
		AA	34 (8.2)	12 (6.5)	1			
		GA	184 (44.3)	86 (46.2)	1.36	0.68–2.73	0.39	
		GG	197 (47.5)	88 (47.3)	1.22	0.59–2.52	0.60	
rs10411210	19q13.11		418 (100)	188				0.83
		TT	11 (2.6)	5 (2.7)	1			
		CT	84 (20.1)	33 (17.6)	0.90	0.25–3.22	0.88	
		CC	323 (77.3)	150 (79.8)	1.05	0.32–3.46	0.93	
rs961253	20p12.3		412 (100)	184				0.29
		CC	202 (49.0)	94 (51.0)	1			
		CA	164 (39.8)	75 (40.8)	1.00	0.62–1.63	0.99	
		AA	46 (11.2)	15 (8.2)	0.64	0.35–1.16	0.14	
rs4925386	20q13.33		413 (100)	182				0.10
		TT	43 (10.4)	18 (9.9)	1			
		TC	171 (41.4)	83 (45.6)	1.15	0.61–2.17	0.66	
		CC	199 (48.2)	81 (44.5)	0.77	0.39–1.51	0.44	

^a^Due to the low frequency, the CC genotype of rs16892766 could not be assessed; the CC and CA genotypes were combined for this SNP

For rs16892766, carriage of the C allele showed a trend of association with a more severe phenotype (OR 1.71, 95 % CI 1.05–2.76, *p* = 0.03, dominant model). At 11q23.1 (rs3802842), a borderline association was observed in the codominant inheritance model (Wald 2df *p* value =0.02), and when tested for the recessive and dominant models of inheritance, carriers of the risk allele of this SNP were also more frequent in the ≥100 polyp group (OR 1.51, 95 % CI 1.03–2.22, *p* = 0.04, dominant model). The other SNPs showed no associations.

When the joint association of the two SNPs (rs16892766 and rs3802842) was tested, both remained borderline significant using dominant mode of inheritance (*p* = 0.04 and *p* = 0.03, respectively), however the interaction of the two SNPs was not significant (*p* = 0.80).

When the total number of sporadic CRC risk alleles in individuals of both groups was compared, the mean number of risk alleles was similar (mean of 13.11 risk alleles for the <100 and 12.90 for the ≥100 group).

## Discussion

In this study, we examined the role of CRC-associated SNPs in disease phenotype in *APC* mutation carriers. Although a correlation between the mutation site in the *APC* gene and the phenotype of FAP is well-established [2], the phenotypic variability observed in patients with the same underlying gene defect suggests that other factors must play a role in modifying disease expression in *APC* mutation carriers. The role of modifier genes in disease severity in FAP patients has been investigated and several modifiers, such as N-acetyl transferases, have been suggested [16–19].

In recent years, several SNPs have been identified that influence CRC risk in the general population. In this study, we investigated whether these SNPs influence the phenotype of patients carrying a pathogenic *APC* mutation. Two variants were found to be associated with the disease phenotype: under a dominant inheritance model, the C alleles of both rs16892766 and rs3802842 showed a trend of association with a phenotype of more than 100 adenomas.

A previous study demonstrated that individuals carrying the risk (C) allele of rs16892766 (8q23.3) present with a more advanced stage of CRC at diagnosis [20]. Tomlinson et al. found that the risk allele of rs16892766 was associated with CRC in younger individuals [4]. In other studies, the risk allele of rs16892766 correlated with an increased CRC risk and/or age of CRC diagnosis in Lynch syndrome [12–14]. In our study, the C allele of this SNP was associated with a more severe FAP phenotype (≥100 polyps) in *APC* mutation carriers. The higher polyp number associated with the C allele of rs16892766 could be explained by the location of this SNP in the *EIF3H* gene, which increases cell proliferation, growth, and survival when overexpressed. However, Carvajal-Carmona et al. [21] suggested that *UTP23*, rather than *EIF3H*, is the most likely target of the genetic variation associated with CRC in the 8q23.3 region, but also proposed that both of these genes may play a role in CRC development, given that they have related roles in mRNA translation. *UTP23* is thought to be involved in ribosome biogenesis [22].

The risk allele of rs3802842 (11q23.1) has been associated with early-onset CRC (<50 years old) and a family history of CRC [20, 23]. Moreover, this SNP is also known to be associated with increased CRC risk in patients with Lynch syndrome [12–14]. A recent study described the association of rs3802842 with disease in patients with unexplained polyposis [20, 24]. In the present study, rs3802842 showed a borderline association with the more severe phenotype of ≥100 polyps in the codominant model of inheritance with two degrees of freedom. When this SNP was tested under recessive and dominant inheritance models, a trend of association was observed between risk allele carriage and the ≥100 polyp phenotype (dominant inheritance model). Functionally, rs3802842 is located within a gene-rich region of chromosome 11q23 that includes four open reading frames (ORFs) within 100 kb: *COLCA1*, *COLCA2*, *POU2AF1* and *C11orf53* (6). The exact function of this SNP is still unknown; one study assessed whether rs3802842 might have cis-regulatory effects on these neighbouring genes, but found no evidence for a relationship. These authors suggested that the underlying sequence change defined by this SNP might exert regulatory effects on genes mapping outside 11q23.1 [25]. Another study suggested that rs3802842 is not itself a functional SNP but is in linkage disequilibrium with a functional SNP [26].

SNPs associated with CRC susceptibility could increase CRC risk by promoting initiation of adenoma formation or promoting growth and/or progression from the adenoma to carcinoma stage, or be involved in both. Theoretically, initiation-promoting SNPs are expected to be more frequent in patients with multiple adenomas and in CRC-free patients with adenoma. A recent study found eight known CRC-associated SNPs, including rs3802842, to be overrepresented in CRC-free patients with adenoma [27]. In relation to the effect of SNPs on the above-mentioned stages, only the association of a CRC-associated SNP at 8q24.21 (rs6983267) with adenoma multiplicity and the association of rs3802842 and rs4779584 with unexplained polyposis have been described to date [6, 24]. Based on these literature reports and the outcome of our study, we hypothesize that rs3802842 is involved in the initiation stage of adenoma development.

An association between the total number of CRC-associated risk alleles and familial CRC has been suggested in two previous studies [28, 29]. Therefore, we investigated whether there was a difference in total number of risk alleles between the two groups. We found the mean number of risk alleles to be similar in the two groups.

Recently, one study examined the severity of polyposis in 64 patients and found no evidence of association in any of their tested SNPs [30], however as stated by Talseth-Palmer et al. [31] large cohorts are required to examine the role of modifiers in severity of disease phenotype in FAP patients.

In conclusion, we identified two CRC-associated SNPs, rs16892766 (8q23.3) and rs3802842 (11q23.1), which show an association with adenoma number in *APC* mutation carriers. In order to evaluate and confirm the effect of these SNPs on the phenotype of FAP, further studies with larger sample sizes are now recommended.
